# Intelligent Monitoring? Assessing the ability of the Care Quality Commission's statistical surveillance tool to predict quality and prioritise NHS hospital inspections

**DOI:** 10.1136/bmjqs-2015-004687

**Published:** 2016-04-18

**Authors:** Alex Griffiths, Anne-Laure Beaussier, David Demeritt, Henry Rothstein

**Affiliations:** 1 School of Management & Business, King's College London, London, UK; 2 Department of Geography, King's College London, London, UK

**Keywords:** Quality improvement methodologies, Risk management, Quality measurement, Performance measures, Health policy

## Abstract

**Background:**

The Care Quality Commission (CQC) is responsible for ensuring the quality of the health and social care delivered by more than 30 000 registered providers in England. With only limited resources for conducting on-site inspections, the CQC has used statistical surveillance tools to help it identify which providers it should prioritise for inspection. In the face of planned funding cuts, the CQC plans to put more reliance on statistical surveillance tools to assess risks to quality and prioritise inspections accordingly.

**Objective:**

To evaluate the ability of the CQC's latest surveillance tool, *Intelligent Monitoring* (IM), to predict the quality of care provided by National Health Service (NHS) hospital trusts so that those at greatest risk of providing poor-quality care can be identified and targeted for inspection.

**Methods:**

The predictive ability of the IM tool is evaluated through regression analyses and χ^2^ testing of the relationship between the quantitative risk score generated by the IM tool and the subsequent quality rating awarded following detailed on-site inspection by large expert teams of inspectors.

**Results:**

First, the continuous risk scores generated by the CQC's IM statistical surveillance tool cannot predict inspection-based quality ratings of NHS hospital trusts (OR 0.38 (0.14 to 1.05) for Outstanding/Good, OR 0.94 (0.80 to −1.10) for Good/Requires improvement, and OR 0.90 (0.76 to 1.07) for Requires improvement/Inadequate). Second, the risk scores cannot be used more simply to distinguish the trusts performing poorly—those subsequently rated either ‘Requires improvement’ or ‘Inadequate’—from the trusts performing well—those subsequently rated either ‘Good’ or ‘Outstanding’ (OR 1.07 (0.91 to 1.26)). Classifying CQC's risk bandings 1-3 as high risk and 4-6 as low risk, 11 of the high risk trusts were performing well and 43 of the low risk trusts were performing poorly, resulting in an overall accuracy rate of 47.6%. Third, the risk scores cannot be used even more simply to distinguish the worst performing trusts—those subsequently rated ‘Inadequate’—from the remaining, better performing trusts (OR 1.11 (0.94 to 1.32)). Classifying CQC's risk banding 1 as high risk and 2-6 as low risk, the highest overall accuracy rate of 72.8% was achieved, but still only 6 of the 13 Inadequate trusts were correctly classified as being high risk.

**Conclusions:**

Since the IM statistical surveillance tool cannot predict the outcome of NHS hospital trust inspections, it cannot be used for prioritisation. A new approach to inspection planning is therefore required.

## Introduction

As England's independent quality regulator for health and social care, the Care Quality Commission (CQC) uses bespoke statistical surveillance tools to monitor care quality and ‘predict where there may be problems and make better decisions about when, where and what to inspect’.^1^ These tools exploit the wealth of administrative and outcome data generated by the NHS to identify those providers at greatest risk of providing poor-quality care so that the CQC can prioritise them for inspection and avoid using its limited resources to check up on trusts providing good-quality care.^2–4^ The UK government now requires all regulators to follow this ‘risk-based’ approach to targeting their inspections in order to ease administrative burdens on regulatees and ensure the proportionality of enforcement action.^5–7^ In addition to serving the government's ‘better-regulation’ goals, risk-based prioritisation is also important for the CQC itself, given the vast number of health and social care providers its c.1400 inspectors are responsible for overseeing; currently, some 30 261 registered providers operating at 49 528 different locations across England.^8–10^ With the government reportedly now contemplating funding cuts of up to 40% to NHS inspectorates, the CQC is going to become increasingly reliant on statistical surveillance tools to help it plan and prioritise its inspections according to risk.^11^ ^12^

The use of statistical surveillance tools to assess performance and prioritise NHS hospital trusts for inspection is well established. The first system of its kind developed by the CQC's predecessor, the Healthcare Commission, showed early signs of success.^13^ However, over the past decade inspections, the standards they assess, and the data available to prioritise them have changed significantly. Developed by the management consultants *McKinsey & Company*,^14^ the CQC's current statistical surveillance tool is called *Intelligent Monitoring* (IM) and is far simpler than earlier tools. IM generates a single trust-level ‘risk score’ based on approximately 150 performance indicators for each NHS trust.^15^ All of this information is published by the CQC but at present does not fully direct the CQC's inspection activity as it has committed to inspecting and rating all trusts by July 2016, regardless of the risk of them providing poor-quality care.^9^ Once this initial baseline round of inspection-based quality rating is complete, however, the CQC has said it then plans to use statistical surveillance to help it identify which hospital trusts should be targeted for further inspection and improvement.^11^ With funding cuts likely to reduce the number of inspections the CQC can conduct, accurate targeting will become even more important. Recently, however, concerns have been raised about the quality and reliability of the indicators used by the IM tool and how they have been combined to calculate the overall risk score.^16–18^ To date, however, there have been no peer-reviewed assessments of the predictive ability of IM.

This study addresses that gap by assessing the ability of the CQC's IM statistical surveillance tool to predict the quality of care provided by NHS hospital trusts and thus identify those that should be prioritised for inspection. Our analysis focuses on the case of NHS hospital trusts because these providers offer the most complete and comprehensive set of performance data for statistical surveillance of any area of health and social care. If the IM tool cannot predict which hospital trusts are most likely to be found wanting by inspectors, then it is likely to face even greater challenges in other areas of health and social care that do not offer the same wealth of performance data for statistical surveillance.

## Method

The aim of this study is to assess the ability of the CQC's IM statistical surveillance tool to predict the quality of care provided by NHS hospital trusts and subsequently prioritise them for inspection. To do so, our analysis measures the statistical relationship between IM risk scores and subsequent quality ratings awarded to NHS hospital trusts following detailed on-site inspections. The datasets and methods for this analysis are detailed below.

### Data

#### Independent variables: quantitative risk scores and ordinal risk bandings

Introduced in 2013, the IM tool uses a simple, unweighted method for aggregating approximately 150 indicators to produce a continuous risk score *R*. These indicators were selected by *McKinsey & Company* and the CQC following a broad consultation process to identify those indicators that are ‘most important for monitoring risks to the quality of care’.^19^ These indicators cover a range of areas including, inter alia, mortality rates, waiting times, whistle-blower reports, staff and patient surveys and ‘Healthcare Worker Flu vaccination uptake’.^15^

Each IM indicator has specific scoring criteria determined by the CQC. For the most part, these scoring criteria are based on a well-established system of Winsorised z-scores^2^ ^13^ ^15^ ^20^ which defines indicator performance either in terms of statistical deviations from the norm or a predefined target.^15^ For each indicator, the IM assigns a trust with one of three ordinal scores:

- 0 (‘No evidence of risk’)
- 1 (‘Risk’)
- 2 (‘Elevated risk’)

Each trust's overall IM risk score, *R*, is then calculated as a percentage by dividing the sum of these individual scores by the worst possible score; that is, two (the score for ‘Elevated risk’) multiplied by the number of indicators that are relevant to the trust.^15^ The greater the value of the risk score *R*, the greater the risk of the trust performing poorly. Risk scores for all trusts were first published on 21 October 2013 and were then simultaneously updated on four subsequent occasions.

The CQC also classifies trusts into one of six ordinal risk bands according to their risk score *R* (table 1).

**Table 1:** Risk bandings applied by CQC to the continuous risk score *R*^15^

Risk score: *R*	Risk band
*R*≥7.0%	Band 1 (highest risk)
5.5%≤*R*<7.0%	Band 2
4.5%≤*R*<5.5%	Band 3
3.5%≤*R*<4.5%	Band 4
2.5%≤*R*<3.5%	Band 5
0≤*R*<2.5%	Band 6 (lowest risk)

There are only two circumstances in which the CQC will manually reclassify a trust from the banding to which it was automatically assigned on the basis of its published risk score *R*, which is itself never adjusted and always represents the pure, quantitative risk score generated by the IM tool. First, trusts placed in ‘special measures’ by the CQC or the economic regulator Monitor—that is, trusts identified as ‘not providing good and safe care to patients’ and whose ‘management cannot fix the problems by themselves’—are classified by the CQC as band 1* regardless of their underlying risk score *R.*^15^ ^21^ Second, trusts that have recently been inspected are automatically banded as ‘Recently inspected’. These manual adjustments to the risk bandings are made by the CQC to avoid any dissonance arising between CQC's considered view, as reflected in the assignment of ‘special measures’ or the inspection-based quality rating, and the risk banding.^22^ Given these manual adjustments, our analysis uses the CQC's continuous risk score *R* and the associated unadjusted risk bandings generated by the IM tool as they represent the pure, forward-looking prediction of the risk of poor-quality care, rather than a post-hoc adjustment of that prediction to reflect a known historical outcome.

#### Dependent variables: inspection-based quality ratings

Inspection-based quality ratings are assigned to individual NHS hospital trusts by the CQC under a new regime of trust-wide inspection introduced in 2013. Under that regime, inspections are conducted by large specialist teams who assess individual hospital services against five ‘key questions’: is the service ‘safe’, ‘effective’, ‘caring’, ‘responsive to people's needs’ and ‘well led’?^22^ Based on their inspection teams’ on-site visits, the CQC then award one of four possible ordinally ranked ratings for each service within a hospital:

1. Outstanding
2. Good
3. Requires improvement
4. Inadequate

The CQC then aggregates those service-level ratings using an algorithm to assign hospital-level ratings, which, in turn, are further aggregated, using a similar algorithm, to generate an overall trust-level rating. These aggregated trust-level ratings, but not the underlying rules for generating them, are publicly available on the CQC website.

Between 17 October 2013, when the new style of inspection began, and 29 September 2015, the CQC completed and published the quality ratings resulting from 103 inspections of 90 different NHS hospital trusts. This represents 55% of all NHS hospital trusts in England. The CQC has committed to inspecting the remaining trusts at least once before July 2016^9^ to provide a baseline against which to measure future performance. Accordingly, in scheduling this initial round of inspections, CQC senior management considered a host of factors in addition to the risk score including the geographical availability of expert inspection team members; the time since the last inspection; the need to evaluate the new inspection methodology; and the commitment to inspect all trusts regardless of risk.

**Figure 1:**
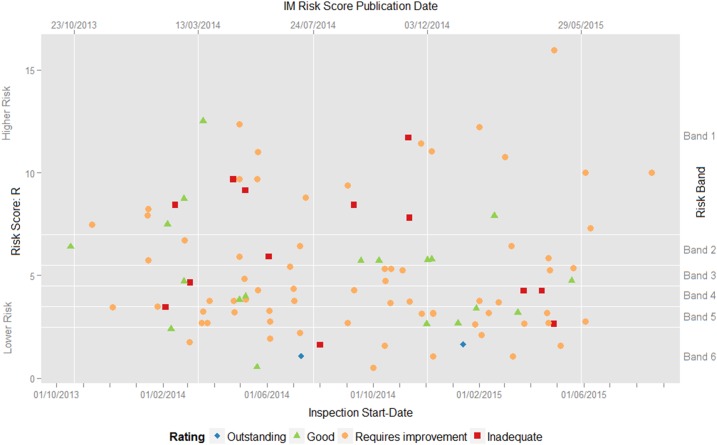
Time series of inspection-based quality ratings (coloured shapes) charted by the most recently published continuous risk score R (left y-axis) and associated ordinal risk banding (right y-axis) at the start of the inspection. The publication date for each of the five sets of Intelligent Monitoring (IM) risk scores generated to date is shown with a white vertical line.

Visual inspection of figure 1 does not highlight any clear temporal trends in the risk scores *R* (left y-axis), risk bandings prior to any adjustment (right y-axis), time elapsed between risk score publication date and inspection start-date (distance from preceding vertical line) or inspection-based quality ratings (shape and colour), each of which might have been indicative of selection bias in our sample of trusts.

The summary statistics in table 2 also support the assumption that our sample is representative. Although there is evidence for some initial prioritisation of trusts in high-risk bands, thereafter the distribution of trusts between high-risk and low-risk bands, and in terms of their subsequent inspection-based quality ratings, is fairly consistent over time. Moreover, there is little variation by risk banding or inspection outcome in the average elapsed time between the publication of the risk scores and the inspection start-date during which the quality of care has the potential to worsen or improve.

**Table 2:** The number of inspections and the average elapsed time (in days) between the inspection start-date and the most recently published Intelligent Monitoring (IM) risk score aggregated by risk banding and inspection-based quality rating

	IM risk score publication date											
	21 October 2013		13 March 2014		24 July 2014		3 December 2014		29 May 2015		Total	
	n	Average time	n	Average time	n	Average time	n	Average time	n	Average time	n	Average time
Risk banding												
High risk (bands 1–3)	11	92.3	13	66.7	11	83.7	12	91.1	3	32.3	50	79.9
Low risk (bands 4–6)	5	98.4	18	62.1	9	78.0	20	80.5	1	5.0	53	74.1
Total	16	94.2	31	64.1	20	81.2	32	84.5	4	25.5	103	76.9
Inspection rating												
Inadequate	3	119.0	3	59.3	4	69.0	3	129.7	0	–	13	92.3
Requires improvement	8	85.0	23	65.7	13	83.5	21	87.2	4	25.5	69	75.5
Good	5	94.0	4	44.3	3	87.3	7	63.3	0	–	19	71.2
Outstanding	0	−	1	119.0	0	−	1	41.0	0	–	2	80.0
Total	16	94.2	31	64.1	20	81.2	32	84.5	4	25.5	103	76.9

There are good reasons, therefore, to assume that the 55% sample of hospital trusts that have so far been inspected is broadly representative of the totality of NHS hospital trusts and provides a robust sample for analysis.

#### Data linkage

We paired each of the 103 trust-level inspection-based quality ratings in our dataset with the most recent risk score generated by the IM tool for the corresponding NHS trust prior to its inspection.

### Statistical methods

We conducted a series of statistical tests to assess the ability of IM to predict the quality of care provided by NHS trusts and identify which ones should be prioritised for inspection. First, we used ordinal regression analysis to test the hypothesis (H1):

(H1): IM risk scores can predict the subsequent inspection-based quality rating

By testing the statistical relationship between the inspection-based quality ratings and the continuous risk score, *R*, ordinal regression makes full use of all the information to assess the ability of IM to predict the quality of care provided by NHS hospital trusts. This ability to predict the probability of any given inspection-based quality rating represents the ideal for a fully risk-based approach to prioritisation. If the CQC could predict the specific quality rating, it would then be able to set differentiated thresholds for triggering inspections that reflect how its tolerance for false positive errors (inspecting trusts performing well) and false negative errors (not inspecting trusts performing badly) may vary depending upon how bad performance is likely to be.

For the purposes of prioritisation, however, predicting the exact inspection-based quality rating may not be necessary. Statistical surveillance could still be useful so long as IM can distinguish trusts performing acceptably from those performing below some threshold of acceptability. To assess its ability at this less demanding task, we conducted two further sets of statistical tests. First, we conducted a series of binary logistic regressions to see whether the continuous risk score *R* could distinguish between different acceptability thresholds. Second, we used the CQC's unadjusted ordinal risk bandings, rather than the continuous risk score *R*, to divide trusts into ‘high-risk’ and ‘low-risk’ groups for which we then ran a series of χ^2^ tests of independence to see if there is any statistically significant association between those binary groupings and various possible thresholds of acceptability in the subsequent inspection-based quality ratings. This latter approach to assessing the value of statistical surveillance has been adopted by the CQC itself and National Audit Office (NAO).^23^

These two statistical tests were both run to test two further hypotheses about IM and its ability to identify trusts that should be prioritised for inspection:

(H2) IM can distinguish the trusts performing poorly—that is, those subsequently rated either ‘Requires improvement’ or ‘Inadequate’—from the trusts performing well—that is, those subsequently rated either ‘Good’ or ‘Outstanding’.

(H3) IM can distinguish the trusts rated ‘Inadequate’—the most serious category of poor performance—from all other trusts.

Following this same logic, we might also have tested whether IM can successfully predict which trusts would be rated ‘Outstanding’, and should therefore be excluded from any inspections. However, with only two out of 103 trusts inspected to date having been rated ‘Outstanding’, this hypothesis could not be robustly assessed.

For each of the three hypotheses, we report the key findings in the next section of the paper and confine the more technical statistical output to appendices. Since these analyses involve only one independent variable—either the continuous risk score, *R*, or some binary grouping of the data based upon it—there are no issues with multicollinearity.

## Results

**(H1): IM risk scores can predict the subsequent inspection-based quality rating**

**Figure 2:**
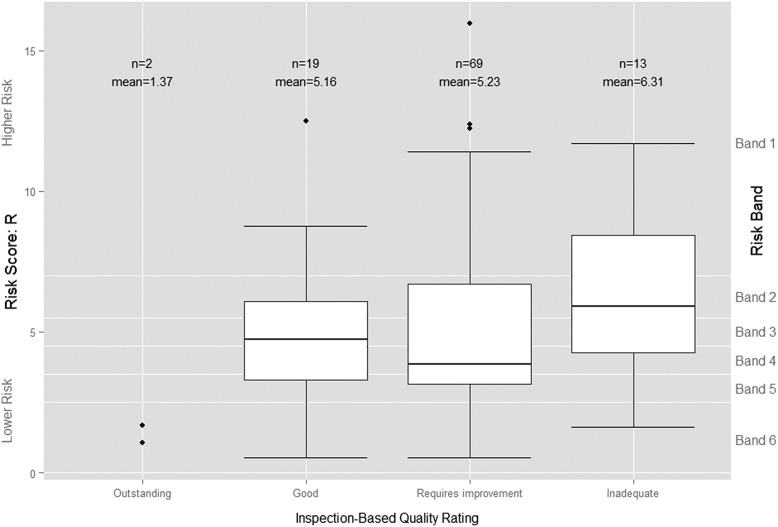
Boxplot of Intelligent Monitoring (IM) risk scores by Care Quality Commission's (CQC's) subsequent inspection-based quality rating. The bottom line (or whisker) indicates the lowest value of R, the bottom edge of the white box indicates the first quartile, the line in the middle of the box the median and the line at the top of the white box the third quartile. The top line (or whisker) indicates the maximum value of R or 1.5 times the interquartile range, if that is less than the maximum value of R, with outlying values of R indicated by individual points.

Figure 1 above does not suggest any clear relationship between the continuous risk scores and the subsequent inspection-based quality ratings. Visual comparison of the distribution of IM risk scores by subsequent rating does not suggest a clear relationship either (figure 2). While the mean risk score for the two trusts rated ‘Outstanding’ is much lower than those for the other three categories, a number of other trusts had similar, or even lower, risk scores than the two rated ‘Outstanding’, including one given the very lowest rating of ‘Inadequate’. Similarly, the predictive ability implied by the upward trend in the mean risk scores for each successively more negative category of inspection-based quality rating is belied by the substantial overlap between their risk score distributions. Indeed, the median risk score, which is less influenced by outliers and provides a better measure of central tendency than the mean risk score, was actually higher for those trusts subsequently rated ‘Good’ (*R*=4.74%), than for those trusts rated ‘Requires improvement’ (*R*=3.85%).

The ordinal logistic regression model confirms our initial visual assessment of the data. Table 3 above indicates that the risk score *R* is significant at the 10% level when differentiating between the ‘Good’ and two ‘Outstanding’ trusts—the least useful distinction in a risk-based approach—but not between any of the other ordinal quality ratings (see online supplementary appendix A for further details).

**Table 3:** The regression coefficients and associated SEs, ORs and associated 95% CIs and p values for the ordinal logistic regression

	Beta (SE)	95% CI for OR			
		2.50%	OR	97.50%	Pr(>|z|)
Outstanding/good (intercept)	−1.190 (0.898)				
Good/requires improvement (intercept)	−1.023 (0.468)				
Requires improvement/inadequate (intercept)	2.520 (0.600)				
Outstanding/good, R	−0.965 (0.519)	0.138	0.381	1.053	0.063
Good/requires improvement, R	−0.066 (0.081)	0.799	0.936	1.097	0.416
Requires improvement/inadequate, R	−0.103 (0.085)	0.764	0.902	1.066	0.228

10.1136/bmjqs-2015-004687.supp1 Supplementary data

The *IM* risk scores therefore are not statistically significant predictors of inspection outcomes. This finding, coupled with the distribution of risk scores preceding each inspection rating, suggests that IM cannot predict inspection-based quality ratings.

**(H2) IM can distinguish the trusts performing poorly—that is, those subsequently rated either ‘Requires improvement’ or ‘Inadequate’ from the trusts performing well—that is, those subsequently rated either ‘Good’ or ‘Outstanding’.**

If the IM tool is not able to predict specific inspection-based quality ratings, it might still be useful for prioritising inspections if it can at least predict which trusts are performing poorly, regardless of the severity of that poor performance, so that they can be prioritised for inspection. To test that hypothesis, we combined those trusts rated ‘Requires improvement’ and ‘Inadequate’ to form a new ‘performing poorly’ category and compared them against those rated ‘Outstanding’ and ‘Good’, which were combined to form a new ‘performing well’ category.

**Figure 3:**
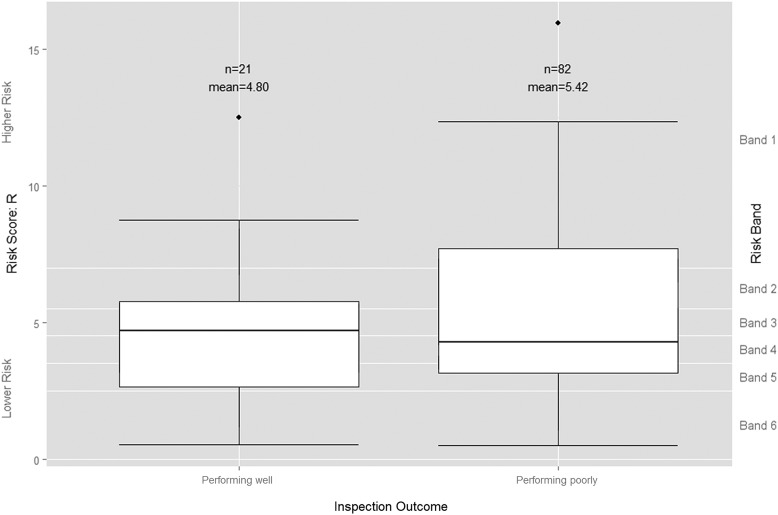
Boxplot of Intelligent Monitoring risk scores by grouped inspection-based quality rating. Trusts rated by the Care Quality Commission (CQC) as ‘Outstanding’ or ‘Good’ are grouped here as ‘Performing well’, while those the CQC rated as ‘Requires improvement’ or ‘Inadequate’ are grouped as ‘Performing poorly’.

Visual comparison of the distribution of the risk scores by these two new inspection-based quality categories suggests that the risk score *R* cannot distinguish those trusts performing poorly from those performing well (figure 3). While the mean risk score for trusts subsequently judged to be ‘performing poorly’ is greater than for trusts judged to be ‘performing well’, the median risk score is *lower*, indicating a lower probability of poor performance for trusts judged to be ‘performing poorly’ than for those judged to be ‘performing well’.

With a p value of 0.424 and an OR of 1.069 (95% CI 0.908 to 1.257), logistic regression analysis shows that the continuous risk score is not a statistically significant predictor of whether or not a trust is performing poorly (see online supplementary appendix B). The substantial variation in risk scores preceding each rating category resulted in a very ill-fitting model with a pseudo Nagelkerke R^2^ value of 0.026 and a receiver operating characteristic (ROC) area under the curve value of 0.540 (95% CI 0.321 to 0.599). The continuous risk score, therefore, does not provide a reliable basis for identifying those trusts that are performing poorly and should be prioritised for inspection.

If logistic regression shows that the continuous risk score *R* is of no use in predicting poor performance, an alternative approach would be to group the trusts using CQC's unadjusted risk bandings, so as to study the general signal from the IM tool rather than the precise risk scores. We can then use the χ^2^ test of independence to see if these banded risk scores can be used to distinguish trusts that are ‘performing poorly’ from those that are ‘performing well’. That is, can we identify a ‘high-risk’ group in which a significantly greater proportion of trusts are ‘performing poorly’ compared with the ‘low-risk’ group? We tried a number of approaches to using the risk scores to divide trusts into low-risk and high-risk categories. First, following the approach taken by the CQC and NAO, we categorised the CQC's risk bands 1, 2 and 3 as ‘high risk’ and bands 4, 5 and 6 as ‘low risk’ and then compared the subsequent inspection-based quality ratings awarded to trusts in each of our two risk categories (table 4).

**Table 4:** A contingency table showing the number of trusts classified as ‘high risk’ (ie, having a risk score in bands 1, 2 or 3) and ‘low risk’, that is, having a risk score in bands 4, 5 or 6, against their subsequent inspection outcome

	‘Performing well’	‘Performing poorly’
‘High risk’	11	39
‘Low risk’	10	43

The number of incorrect predictions (11 false negatives+43 false positives=54 errors, shaded in red) slightly exceeds the number of correct predictions (39+10=49, shaded in green), resulting in an overall accuracy rate of just 47.57% (49/103).

We tried the four other possible groupings and no cut-off point produces a significant result in the χ^2^ tests (full details are available in see online supplementary appendix B). The *IM* tool, therefore, cannot pass the less onerous test of predicting a high-risk group of trusts that are significantly more likely to be ‘performing poorly’ and that the CQC might be able to prioritise for inspection.

**(H3) IM can distinguish the trusts rated ‘Inadequate’—the most serious category of poor performance—from all other trusts.**

**Figure 4:**
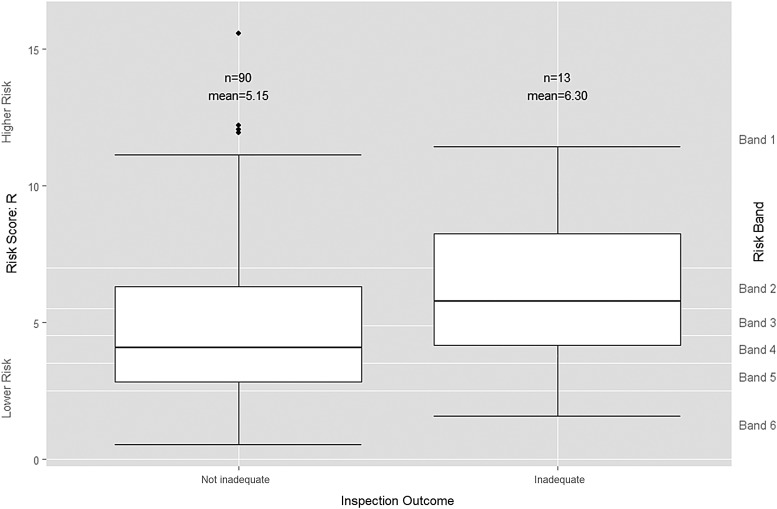
Boxplot of risk scores by grouped inspection-based quality rating. Trusts rated by Care Quality Commission as ‘Outstanding’, ‘Good’ or ‘Requires improvement’ are grouped together as ‘Not inadequate’ while trusts rated as ‘Inadequate’ remain as ‘Inadequate’.

If IM cannot distinguish between those trusts ‘performing poorly’ and those trusts ‘performing well’, it might still be useful for prioritisation if it can at least predict which trusts will be found ‘Inadequate’ in order to target them for immediate investigation. An initial visual assessment of the distribution of risk scores suggests that the risk score has somewhat more ability at predicting whether or not a trust is ‘Inadequate’ (figure 4) than it does for distinguishing the broader category of trusts that are ‘performing poorly’ (figure 3). Both the mean and median risk scores for trusts that were subsequently rated as ‘Inadequate’ are higher than for those trusts that were not.

However, logistic regression for the risk score *R* as the sole independent variable produced an insignificant p value of 0.225 with an OR of 1.111 (95% CI 0.937 to 1.317) (see online supplementary appendix C). The logistic regression model is again a very poor fit with a Nagelkerke pseudo R^2^ score of 0.010 and an ROC area under the curve value of 0.616 (95% CI 0.450 to 0.783). There is so much variation in the risk scores for those trusts subsequently rated as ‘Inadequate’ and those trusts that were not, that no well-fitting binary logistic model exists.

We also examined the banded risk scores to see whether a greater proportion of ‘Inadequate’ trusts could be categorised as ‘high risk’ rather than ‘low risk’. The most statistically significant result using CQC's bandings is achieved when CQC risk band 1 is classified as ‘high risk’ and all the other CQC risk bands are classified as ‘low risk’ (table 5).

**Table 5:** A contingency table showing the number of trusts classified as ‘high risk’ (ie, having a risk score in band 1) and ‘low risk’ (ie, having a risk score in bands 2, 3, 4, 5 or 6) against their subsequent inspection outcome

	‘Not inadequate’	Inadequate
High risk	21	6
Low risk	69	7

This time, the number of correct predictions (6+69=75, shaded in green) far exceeds the number of incorrect predictions (21 false positives+7 false negatives=28, shaded in red), for an overall accuracy rate of 72.82% (75/103). The χ^2^ value is significant at the 10% level (p=0.080). Since the aim of this prioritisation scheme is to focus CQC inspections on those trusts most likely to be ‘Inadequate’, the 20.38% false positive error rate may be less important than the false negative failures of detection. Unfortunately, however, this prioritisation scheme identified less than half (6 out of 13) of the trusts found to be ‘Inadequate’. The results of χ^2^ tests on three possible remaining, less statistically significant classification schemes are detailed in online supplementary appendix C.

The *IM* tool, therefore, was able to categorise trusts as high or low risk with a significantly greater proportion of high-risk trusts subsequently being found ‘Inadequate’. The practical benefit of such a prioritisation method is questionable, however, as it successfully identifies fewer than half of the ‘Inadequate’ trusts, incorrectly identifies more than three times as many ‘Not Inadequate’ trusts and does not account for ‘Requires improvement’ trusts which are also performing poorly. The CQC, understandably, has a very limited risk appetite and no tolerance for ‘Inadequate’ care.^24^

## Discussion

### Statement of principal findings

The IM tool failed three successively less demanding statistical tests of its ability to predict quality and identify which NHS hospitals should be prioritised for inspection. First, ordinal logistic regression confirmed that the IM tool's risk scores cannot predict inspection-based quality ratings of NHS hospital trusts. Statistical surveillance does not provide the CQC with a statistically significant basis for setting differentiated thresholds for triggering inspections that reflect how its tolerance for false positive errors (inspecting trusts performing well) and false negative errors (not inspecting trusts performing badly) may vary depending upon how bad performance is likely to be.

Second, the risk scores cannot be used more simply to distinguish the trusts performing poorly—that is, those subsequently rated by inspectors either ‘Requires improvement’ or ‘Inadequate’—from the trusts performing well—that is, those subsequently rated either ‘Good’ or ‘Outstanding’. The binary logistic regression results in a p value of 0.424 indicating no statistically significant relationship between the continuous risk score and the subsequent performance category. Furthermore, the risk scores cannot be used to perform the less onerous task of determining a high-risk group of trusts which contain a significantly greater proportion of trusts ‘performing poorly’ than in the low-risk group containing the remaining trusts.

Finally, the risk scores are of limited use for distinguishing the worst performing trusts—that is, those subsequently rated ‘Inadequate’—from the rest. For this test, the binary logistic regression results in an insignificant p value of 0.225. It is possible to use the risk scores to define a high-risk group of trusts of which 6 out of 27 are ‘Inadequate’; a significantly greater proportion than in the low-risk group of trusts of which 7 out of 76 are ‘Inadequate’. Although this classification is statistically significant at the 10% level (p=0.080), its practical value for prioritisation is limited as it successfully identifies fewer than half of the ‘Inadequate’ trusts that must be prioritised for inspection.

### Strengths and limitations of the study

Our analysis includes all 103 inspections completed and published in the 2 years since the launch of the new IM statistical surveillance tool. This dataset includes inspections of just over half of all NHS hospital trusts in England. The sample showed little evidence of significant temporal bias or trend in terms of risk score, risk bandings, time elapsed between risk score publication date and inspection start-date, and inspection-based quality rating.

One limitation of the study is the possibility that the CQC may have used accurate supplementary intelligence either to bring forwards the inspection of some trusts with low-risk scores that it suspects, for reasons not reflected by the IM, of delivering poor care or to delay the inspection of some trusts with high-risk scores that it suspects, for reasons outside of IM, of delivering good care. This would increase or decrease respectively the probability of an inaccurate risk score being included in our sample, and could understate or overstate the ability of IM. Inaccurate supplementary intelligence may have the opposite effect. It is not possible to determine the extent or direction of this possible bias.

### Policy implications

This study highlights problems with the CQC's risk-based approach to making ‘sure health and social care services provide people with safe, effective, compassionate, high-quality care’.^25^ This regulatory strategy depends on the ability of the CQC to predict which providers are at greatest risk of performing poorly so that it can then prioritise them for inspection and improvement. Yet our analysis shows that the CQC's current surveillance tool—*IM*—is unable to predict which hospital trusts are most likely to be providing poor-quality care. By the CQC and NAO's measure, IM's predictions are wrong more often than they are right.

Potential funding cuts and other pressures, however, have led the CQC to announce plans for greater prioritisation of its future inspection activities according to risk.^11^ If this is to be the case, then a new statistical surveillance approach will be required. Given the nature of our data and analysis, we cannot say why the risk scores produced by the CQC's IM statistical surveillance tool failed to predict the inspection-based quality ratings. There are, however, a number of possible explanations that should be investigated in developing an improved approach.

It may be that the IM tool is too simplistic. Some IM indicators may be more effective predictors of care quality than others, but their signal could be going undetected by the tool because it weights each indicator equally. Moreover, the NHS generates hundreds of available indicators that are not currently used by the IM tool, but which may be more effective predictors of care quality. An ideal next step, therefore, would be to conduct a machine learning exercise to determine whether there is any combination of indicators that could predict the outcome of historic inspections more reliably. The development of a successful model would offer hope for the continued use of a risk-based approach.

Should it prove impossible to develop an improved model through machine learning, it may be because existing indicator data are inadequate. For example, IM can only produce trust-level risk scores as the majority of data is only available at trust level. This may be too coarse in scale to discern the localised pockets of poor quality detected by skilled inspectors. The collection of more hospital-level data might resolve this problem but the cost and practicality might be prohibitive.

If gathering improved data and then using a machine learning approach does not work, there would then be two further possible explanations for why the current, or indeed any future improved statistical surveillance model, finds it difficult to predict the outcome of CQC inspections. First, it may be that statistical surveillance is effective at identifying poor-quality care, but that the quality ratings assigned by inspectors are unreliable.^26^ However, the current system of CQC's comprehensive trust-wide inspections by large teams of specialist inspectors, clinicians and ‘experts by experience’ have received widespread support and it would be challenging to expand them further.^8^ ^23^ ^27^ Second, it may be that data-driven statistical surveillance systems and inspectors are simply assessing different things. There are substantial challenges to comprehensively assessing the quality of care provided by large, complex, multisite NHS hospital trusts and their highly skilled workforce. Moreover, judgements of care quality are subjective, intangible and difficult to capture by indicators alone. Indeed, if it were simple, there would be little need for inspection.

Regardless of the explanation, if a reliable method of statistical surveillance cannot be found, then the CQC cannot realise the efficiencies promised by prioritising its inspections according to risk. Moreover, given the prevalence of poor-quality care, risk-based targeting of inspections may provide little benefit. Of the 103 inspections in our sample, 81 resulted in an overall rating of either ‘Requires improvement’ or ‘Inadequate’ and a further 12 included at least one rating of ‘Requires improvement’ concerning whether the trust was ‘safe’, ‘caring’, ‘responsive’, ‘effective’ or ‘well led’. In that context, rather than trying and failing to target inspections, it may be preferable to continue systematically inspecting each trust or adopting a random approach to signal to managers and the public alike that every hospital matters.^28^ With reduced resources, the length of time between inspections is likely to increase, but this may still be more desirable than poor-quality care going undetected for even longer periods in trusts the CQC mistakenly believes to be low risk. Whatever strategy is adopted, there is clear value in empirically evaluating whether the purported benefits are achieved in practice.

### Unanswered questions and future research

While statistical surveillance tools were first developed for monitoring hospitals, the CQC now uses them across the whole health and social care sector. Given the results of our analysis, it would be valuable to assess their effectiveness at identifying poor-quality care in other areas of health and social care and prioritising the CQC's associated inspection activities.

## Conclusions

We have shown that the CQC's *IM* statistical surveillance tool cannot predict which NHS hospital trusts are at greatest risk of delivering poor-quality care and should be prioritised for inspection. A new approach to statistical surveillance and inspection planning is therefore required.
