# Supplementary material for: Intelligent Monitoring? Assessing the ability of the Care Quality Commission's statistical surveillance tool to predict quality and prioritise NHS hospital inspections
Source: BMJ Qual Saf. 2016 Apr 18;26(2):120–30. doi: 10.1136/bmjqs-2015-004687 (PMC5284345; doi:10.1136/bmjqs-2015-004687)
Supplement: Supplementary data [file bmjqs-2015-004687supp.pdf]

Appendix A

Ordinal logistic regression goodness-of-fit test

The goodness-of-fit test proposed by Fagerland, Hosmer and Bofin for multinomial and ordinal logistic regression has a test statistic of $\hat{C}_M = 14.17299$ [1, 2]. If the model is a good fit the test statistic should follow a chi-squared distribution with 24 degrees of freedom (10 groups - 2 multiplied by 4 possible outcomes - 1). Visual inspection shows the model to be a reasonable fit and a p-value of 0.943 confirms this.

Group	Outstanding		Good		Requires improvement		Inadequate		Sum
	Obs.	Est.	Obs.	Est.	Obs.	Est.	Obs.	Est.	
1	2	0.90	1	1.59	6	6.67	1	0.84	10
2	0	0.31	3	2.27	7	7.39	1	1.03	11
3	0	0.17	1	2.11	9	6.74	0	0.99	10
4	0	0.11	1	2.12	8	6.75	1	1.03	10
5	0	0.07	2	2.31	7	7.43	2	1.19	11
6	0	0.03	2	2.04	7	6.76	1	1.18	10
7	0	0.01	4	1.97	5	6.76	1	1.26	10
8	0	0.00	3	1.83	6	6.73	1	1.44	10
9	0	0.00	1	1.81	6	7.32	4	1.87	11
10	0	0.00	1	1.41	8	6.43	1	2.15	10
Total	2	1.60	19	19.45	69	68.97	13	12.98	103

Table 6: Observed (Obs) and estimated expected (Est) frequencies for each rating using the fitted ordinal logistic regression model.

The difficulty with this model, however, is that the outcome with the greatest predicted probability is always 'Requires improvement' and always with a probability greater than 0.5. With 69 out of 103 trusts rated as 'Requires improvement' that means the model is often right but of limited use when looking to distinguish trusts and prioritise them for inspection. All trusts are predicted as being rated the same.

Appendix B

Binary logistic regression model

	Beta (SE)	95% CI for Odds Ratio (OR)			
		2.50%	OR	97.50%	Pr(>|z|)
(Intercept)	1.023 (0.476)	1.095			
Risk Score: R	0.066 (0.083)	0.908	1.069	1.257	0.424

Table 7: The regression coefficients and associated standard errors, odds-ratios and associated 95% confidence intervals, and the p-values for the binary logistic regression for trusts 'performing poorly' or 'performing well'.

The model is a very poor fit with a null deviance of 78.10, residual deviance of 76.69, and a pseudo-Nagelkerke R^2 value of 0.026. The poor fit is confirmed by the ROC curve with an AUC value of 0.540. As with the ordinal logistic regression model used to assess H1, the model predicts all trusts will be 'performing poorly' with a probability greater than 0.5 regardless of their risk score *R*.

Chi-squared tests

For each grouping the chi-squared statistic and associated p-value is shown in the first row. The second row details the sensitivity, or detection rate: the proportion of all Trusts who were judged to be ‘performing poorly’ that were in the ‘high risk’ group and therefore would have been prioritised for inspection. The third row details the specificity, or the proportion of Trusts judged to be ‘performing well’ that were in the ‘low risk’ category and therefore correctly not prioritised. Finally, the fourth row details the proportion of trusts incorrectly prioritised (false positives), that is the proportion of Trusts in the ‘high risk’ category who were subsequently judged to be ‘performing well’.

		Low Risk Group				
Prioritising Trusts 'Performing Poorly'		Bands 2-6	Bands 3-6	Bands 4-6	Bands 5-6	Band 6
High Risk Group: Band 1	Chi-squared value, p-value	0.700, p=0.403				
	Detection rate	23/82 = 28.05%				
	Correctly not prioritised	17/21 = 80.95%				
	Incorrectly prioritised	4/27 = 14.81%				
High Risk Group: Bands 1-2	Chi-squared value, p-value		0.280, p=0.597			
	Detection rate		30/82 = 36.59%			
	Correctly not prioritised		12/21 = 57.14%			
	Incorrectly prioritised		9/30 = 30.00%			
High Risk Group: Bands 1-3	Chi-squared value, p-value			0.155, p=0.693		
	Detection rate			39/82 = 47.56%		
	Correctly not prioritised			10/21 = 47.62%		
	Incorrectly prioritised			11/50 = 22.00%		
High Risk Group: Bands 1-4	Chi-squared value, p-value				0.016, p=0.898	
	Detection rate				52/82 = 63.41%	
	Correctly not prioritised				8/21 = 38.10%	
	Incorrectly prioritised				13/65 = 20.00%	
High Risk Group: Bands 1-5	Chi-squared value, p-value					0.668, p=0.414
	Detection rate					72/82 = 87.80%
	Correctly not prioritised					4/21 = 19.05%
	Incorrectly prioritised					17/89 = 19.10%

Table 8: Chi-squared test assessing whether a whether a significantly greater proportion of trusts ‘performing poorly’ could be identified in a ‘high risk’ group than in a ‘low risk’ group using different CQC bandings to determine the threshold for the ‘high risk’ group.

Appendix C

Binary logistic regression model

	Beta (SE)	95% CI for Odds Ratio (OR)			
		2.50%	OR	97.50%	Pr(>|z|)
(Intercept)	-2.535 (0.609)				
Risk Score: R	0.105 (0.087)	0.937	1.111	1.317	0.225

Table 9: The regression coefficients and associated standard errors, odds-ratios and associated 95% confidence intervals, and the p-values for the binary logistic regression for trusts rated 'Inadequate' or not.

The model is a very poor fit with a null deviance of 104.18, residual deviance of 103.51 and a pseudo-Nagelkerke R^2 value of 0.010. The poor fit is confirmed by the ROC curve with an AUC value of 0.616. Similar to the ordinal logistic regression model used to assess H2, the model predicts all trusts will be in the category that contains 'Requires improvement', in this case 'not Inadequate' with a probability greater than 0.5 regardless of their risk score *R*.

Chi-squared tests

Each row is as described in Appendix B.

			Low Risk Group			
	Prioritising 'Inadequate' Trusts		Bands 2-6	Bands 3-6	Bands 4-6	Bands 5-6
High Risk Group	Band 1	Chi-squared value, p-value	3.058, p=0.0.080			
		Detection rate (sensitivity)	6/13 = 46.15%			
		Correctly not prioritised (specificity)	69/90 = 76.67%			
		Incorrectly prioritised	21/27 = 77.78%			
	Bands 1-2	Chi-squared value, p-value		1.615, p=0.204		
		Detection rate (sensitivity)		7/13 = 53.85%		
		Correctly not prioritised (specificity)		48/90 = 53.33%		
		Incorrectly prioritised		32/39 = 82.05%		
	Bands 1-3	Chi-squared value, p-value			1.006, p=0.316	
		Detection rate (sensitivity)			8/13 = 61.54%	
		Correctly not prioritised (specificity)			58/90 = 64.44%	
		Incorrectly prioritised			42/50 = 84.00%	
	Bands 1-4	Chi-squared value, p-value				1.220, p=0.269
		Detection rate (sensitivity)				10/13 = 76.92%
		Correctly not prioritised (specificity)				35/90 = 38.89%
		Incorrectly prioritised				55/90 = 61.11%

Table 10: Chi-squared test assessing whether a whether a significantly greater proportion of 'Inadequate' trusts could be identified in a 'high risk' group than in a 'low risk' group using different CQC bandings to determine the threshold for the 'high risk' group.

A fifth test where bands 1-5 were classed as 'high risk' and band 6 as 'low risk' was not performed as this would have involved two of the four cells in the chi-squared contingency table having values less than 5 and therefore rendering the test invalid.

References

1. Fagerland MW, Hosmer DW, Bofin AM. Multinomial goodness-of-fit tests for logistic regression models. Statistics in medicine. 2008;27(21):4238-53.
2. Hosmer Jr DW, Lemeshow S, Sturdivant RX. Applied logistic regression: John Wiley & Sons; 2013.
